# Assessment of properties of gluten‐based edible film formulated with beeswax and DATEM for hamburger bread coating

**DOI:** 10.1002/fsn3.3242

**Published:** 2023-01-31

**Authors:** Mehrdad Mohammadi, Alaleh Zoghi, Mohammad Hossein Azizi

**Affiliations:** ^1^ Department of Food Technology Research, National Nutrition and Food Technology Research Institute, Faculty of Nutrition Sciences and Food Technology Shahid Beheshti University of Medical Sciences Tehran Iran; ^2^ Department of Food Science and Technology, Faculty of Agriculture Tarbiat Modarres University Tehran Iran

**Keywords:** beeswax, DATEM, sensory evaluation, shelf life, staling

## Abstract

Using edible films and coatings is one of the effective methods of improving the quality of bread. The aim of the present work was the development of gluten‐based films containing lipids to be applied as bread coating, intending to improve quality and delay staleness. In this study, two types of lipids including beeswax and DATEM (diacetyl tartaric ester monoglycerides) were incorporated into gluten film at different levels. The findings showed that inserting both lipids together into gluten for film preparation, weakened the developed films in terms of mechanical and moisture barrier properties. Adding DATEM to the gluten film formulae decreased the elongation at the break and the tensile strength of the film. Using gluten‐beeswax coatings for hamburger bread, compared to gluten‐DATEM coatings, indicated a significant decrease in the hardness and staling feature. Moreover, applying sorbate as a preservative along with the solvents used in the film preparation prevented the growth of mold during the bread shelf life. In conclusion, the findings in this study indicated that the type and levels of lipids added to the edible gluten‐based films and coatings affected the film properties and coated hamburger bread quality, significantly.

## Graphical abstract

The inserting beeswax and DATEM into gluten for film preparation, weakened the developed films in terms of mechanical and moisture barrier properties. Adding DATEM to the gluten film formulae decreased the elongation at the break and the tensile strength of the film. Using gluten‐beeswax coatings for hamburger bread, compared to gluten‐DATEM coatings, indicated a significant decrease in the hardness and staling feature. The type and levels of lipids added affected the film properties and coated hamburger bread quality, significantly.

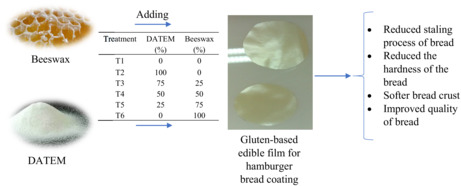

## 1 INTRODUCTION

The majority of the body's daily requirements for energy, minerals, and B vitamins are met by bread, one of the oldest foods consumed by many people worldwide (Geng et al., 2016). Hamburger bread belongs to the group of pan bread. The key ingredients used to make this product are wheat flour, water, yeast, oil, improver, and salt. Yeast is used to prepare the dough for these kinds of bread, and the ideal rest period during bread fermentation and gas evaporation enhances the volume of the dough (Pouya panahi et al., 2022). The wasting of hamburger buns is primarily high due to the low quality, short shelf life, and staleness of this type of bread; therefore, from an economic point of view, it seems necessary to use appropriate methods to increase its quality and shelf life (Mohammadi et al., 2022).

One of the ways to delay the staleness of bread is to prevent the loss of bread moisture by using synthetic packaging films, but the main problem with these packaging is their lack of biodegradation in the environment, and another problem is that after removing the bread from the packaging, the possibility of staleness still exists. One of the most common methods for reducing the rate of staling, increasing the shelf life of bread and consumer acceptance is applying various types of edible films and coatings on the bread surface.

Edible films and coatings are thin layers of edible materials that are placed on the surface of food and are considered as one of the basic ways to control physiological, microbial, and physicochemical changes in food. Edible coatings and films refer to different ideas. While edible coatings are generated directly on the surface of the food products, edible films are thin, solid layers that can be applied to wrap food (Zoghi et al., 2020). Polysaccharides, lipids, and proteins are the three kinds of ingredients that can be employed to create edible films and coatings. The film‐forming solutions may also contain other components, such as plasticizers, to increase their mechanical or structural stability (Valencia‐Chamorro et al., 2011). The main purpose of using edible films and coatings is to prevent the transfer of moisture and oxygen, preserve aromatic substances, flavoring compounds, or oils, improve the quality and appearance and increase the shelf life of food (Silva‐Weiss et al., 2013).

Very high molecular weight, high complexity, and diversity of components are the characteristics of wheat gluten protein that can be used to make edible films and coatings with practical properties. Film formation from wheat gluten solutions has been widely studied (Hernandez–Munoz et al., 2004; Madhu et al., 2020; Shen et al., 2022; Tanada‐Palmu & Grosso, 2003). This process requires the formation and drying of a thin layer of the protein matrix (Wittaya, 2012). The barrier properties of gluten films, such as moisture permeability, can be improved by incorporating a lipid material into their structure (Shen et al., 2022). Diacetyl tartaric ester monoglycerides (DATEM) and beeswax, because of their high moisture retention capacity as hydrophobic substances and their good appearance, are the most effective lipid materials for improving the barrier properties of gluten‐based films (Hammam, 2019). DATAM is an emulsifier that is mainly used in the bakery industry. It is a combination of complex glycerin esters in which one or more hydroxyl groups are esterified with diacetyl tartaric acid and mono or diglyceride. Combining wheat gluten protein with DATEM could reduce water vapor permeability, increase tensile strength, and maintain transparency (Bourtoom, 2009).

Although previous works have demonstrated the enhancing effects of various types of gluten‐based films or coatings for applying to bakery products, investigation regarding the role of lipids, DATEM and/or beeswax, in improving the properties of the gluten‐based films or coatings is not performed yet. This work thus aimed to study the optimum formulation of edible protein‐lipid film and coating and to investigate their use in order to increase the quality of hamburger bread.

## 2 METHODS AND MATERIALS

### 2.1 Materials

Commercial‐type wheat gluten, with the parameters of 4.33% moisture and 80.91% protein, and hamburger bread were purchased from Nanavaran Co. (Tehran, Iran). DATEM was supplied by the enzyme India Company (Karnataka, India). The Beeswax was provided by Fluka company (Buchs, Switzerland). All other chemicals used in this study were obtained from Merck (Darmstadt, Germany).

### 2.2 Film preparation

A suspension of 12.5 g gluten, 20 ml distilled water, and 0.03 g sodium sulfite was prepared. 32 mL ethanol was added after 15 min and the solution was stirred for 10 min at 40°C using a magnetic stirrer (RH‐B2, IKA, Germany). After the addition of 2 ml glycerol, as a plasticizer, the pH of the mixture was adjusted to 5 and the solution was stirred for 10 min using a magnetic stirrer. Then 3 g lipid, including DATEM and/or Beeswax (see Table 1), for each treatment was dissolved and the mixture was heated over the melting point of lipids. Due to the high humidity of the bread surfaces, 0.18 g sorbate (0.01 of the dry matter of the film coating) was added to the solution in this step, in order to prevent mold growth. The final solution was homogenized for 4 min using a homogenizer. Then, the solution was placed on a glass plate (with a thickness of 120 microns) at 25°C and 50% relative humidity for 20 h to be completely dry. Dried films were peeled off the glass petri dishes slowly and maintained in a desiccator at 25°C and 50% relative humidity for 48 h before usage. Water solubility, water vapor permeability, and mechanical properties of the prepared films were measured to select the optimal film.

**TABLE 1:** Composition of gluten‐based^a^ edible film treatments

Treatment	DATEM (%)	Beeswax (%)
T1	0	0
T2	100	0
T3	75	25
T4	50	50
T5	25	75
T6	0	100

Abbreviation: DATEM: Di Acetyl Tartaric Ester of Monoglycerides.

^a^ Each treatment contains 12.5 g of wheat gluten.

#### 2.2.1 Mechanical properties

Mechanical properties of the films, including tensile strength and elongation at break, were determined according to Mohammadi, Azizi, and Zoghi et al. (2020) with some modifications using an Instron Universal Testing Machine (BZ2.5/TH1S, Zwick, Germany) according to the ASTM‐D882‐02. The films were cut into 1.4 × 6 cm pieces and equilibrated at 25°C and 50% relative humidity. Then fixed between the two jaws with an initial distance of 40 mm. Tensile strength was obtained by dividing the peak load in Newton (N) by the cross‐sectional area of the initial film pieces. Elongation at break was calculated by the percentage change in length of the film pieces from 40 mm of the original distance between the jaws.

#### 2.2.2 Water solubility

Water solubility was determined based on the method of Mohammadi et al. (2020). Three circles with a diameter of 2 cm were cut from each film, then the pieces of film were heated in an oven (L3456J, Iran Khodsaz, Iran) at 100°C for 24 h to a constant weight. Then, the pieces of films were immersed in containers containing 50 ml of distilled water and 0.02% (v/w) Sodium azide, to prevent the growth of microorganisms, for 24 h at room temperature on a magnetic stirrer (RH‐B2, IKA, Germany). The films were then filtered to obtain undissolved films and dried at 100°C to reach a constant weight to obtain the final dry weight of the film. By reducing this weight from the weight of the initial dry matter, the weight of the dry matter dissolved in water and its percentage was obtained.

#### 2.2.3 Water vapor permeability

This value was determined using the standard ASTM‐96‐80 method with some modifications. Circular cups with an inner diameter of 3.5 cm, an outer diameter of 5.5 cm, and a depth of 3 cm containing 8 ml of distilled water were sealed by the films with clips and grease. Each cup was placed inside a desiccator with silica gel at 25°C and was weighed every 12 h (using a digital scale with a precision of 0.0001). Water vapor passing through the film and absorbed by the silica gel is obtained by increasing the weight of the silica gel. The relative humidity inside the cup was 100% and the outside of the cup was 0%, which caused a slight partial pressure of 32.23 mmHg between the outside and inside of the cup.

The water vapor permeability was calculated according to the following equation:

$$\text{Permeability}\;\left(\mathrm{gr}.\frac{\mathrm{mm}}{m^{2}}.\mathrm{day}.\text{mmHg}\;\right)=\frac{∆W.X}{A.T.∆P}$$

Where ΔW is weight loss per cup (g); X is film thickness (mm); A is the area of exposure to the cup (9.16 cm^2^); T is time (s); and Δp is the pressure difference on both sides of the film (mmHg).

#### 2.2.4 Color measurement

The color values of films in terms of L* (lightness), a* (redness), and b* (yellowness) were measured using a colorimeter (Cdorflex, USA). Films were placed in desiccators at 25°C and 50% relative humidity for 48 h before measurement (Hernandez–Munoz et al., 2004). Triplicate measurements of color were conducted for each film.

### 2.3 Coating of bread samples

The prepared solution for film production was sprayed onto the whole surface of the fresh hamburger bread samples using a spray device (WALMEC, Asturo///EC, Italy). For this purpose, a 30 ml solution was sprayed evenly on each bread surface with a pressure of 2 bar, and a constant distance of 20 cm. Then the bread samples were immediately placed in an oven (L3456J, Iran Khodsaz, Iran) at 60°C for 30 min to evaporate the solvents and form a coating (Bravin et al., 2006).

### 2.4 Coated bread analysis

#### 2.4.1 Hardness evaluation

Some of the bread samples were placed in polyethylene bags and kept at room temperature, and others were placed without polyethylene bags at 50% relative humidity and 25°C inside a humidifier (IKH.RH, Iran Khodsaz, Iran). The hardness of bread samples was analyzed by a bread hardness tester (H50KS, Hounsfield, UK) according to AACC (2000) No. 74‐09 in the first, third, and fifth days of storage time for samples with polyethylene bags, and in the first, second, and third days of storage time for samples without polyethylene bags.

#### 2.4.2 Sensory evaluation

The staling feature of samples was assessed according to AACC (2000) No. 74‐30, after 1, 3, and 5 days of storage time in Polyethylene bags at room temperature. For this purpose, 25 trained panelists were asked to rate the bread samples in the ranks of 1 to 6 in terms of staleness. So, the freshest bread was given the rank of 6 and the stalest bread was given the rank of 1. The samples were identified with random three‐digit numbers.

#### 2.4.3 Evaluation of the appearance of coated bread in terms of mold growth

Samples in polyethylene bags were evaluated visually for mold growth on the first, third, and fifth day of storage time at 25°C (Plessas et al., 2008).

### 2.5 Statistical analyses

The data were analyzed by one‐way analysis of variance (ANOVA) and comparison of the mean of data with Tukey's test. *p* values <.05 were considered statistically significant. All tests were carried out three times and analyzed using SPSS 16 statistical package (SPSS Inc.). Friedman test was used to evaluate the results of the sensory assessment of bread samples.

## 3 RESULTS AND DISCUSSION

### 3.1 Mechanical properties of the film

The crucial mechanical characteristics of edible films are their tensile strength and elongation at break. To provide effective food safety during circulation and storage, high tensile strength along with low elongation characteristics are important (Tosif et al., 2021). Adding beeswax to the gluten‐based film weakens the film and increases its elasticity, which is due to the diffusion of beeswax particles inside the gluten network and reducing the strength of this network. However, due to the reaction of DATEM with gluten, it does not play a significant role in the resistance of the film to tension, and the tensile strength is significantly reduced due to the strength of the gluten network by the DATEM (Hammam, 2019). As can be seen in Table 2, there is a significant difference in tensile strength between all treatments. The highest tensile strength was observed in treatment 1 (8.96) and followed by treatments 2 and 3. In treatment 4, the lowest tensile strength was seen (3.51). So, in general, it can be said that DATEM has reduced the tensile strength in the gluten‐based film to some extent, but beeswax causes a sharp decrease in tensile strength.

**TABLE 2:** Mechanical properties of gluten‐based edible films

Treatment^A^	Tensile strength (MPa)	Elongation at break (%)	Water solubility (%)	Water vapor permeability (gr.Mm/m^2^.Day.mmHg)
T1	8.96 ± 0.27^a^	60.10 ± 10.31^a^	20.60 ± 0.20^a^	3.25 ± 0.08^a^
T2	8.42 ± 0.22^b^	3.90 ± 0.71^b^	16.07 ± 0.25^b^	2.50 ± 0.15^b^
T3	7.75 ± 0.15^b^	5.84 ± 0.79^b^	15.60 ± 0.10^bc^	1.99 ± 0.12^c^
T4	3.51 ± 0.15^c^	109.00 ± 7.21^c^	15.00 ± 0.20^c^	1.74 ± 0.11^cd^
T5	5.34 ± 0.11	117.00 ± 1.73^c^	13.93 ± 0.51^d^	1.63 ± 0.10^cd^
T6	4.26 ± 0.16^d^	181.00 ± 15.72^d^	13.33 ± 0.15^d^	1.24 ± 0.11^d^

*Note*: Data are expressed as mean values. Values in the columns followed by different lowercase letters are significantly different (*p* < .05).

^A^ For treatment descriptions see Table 1.

It has been reported that increasing beeswax supplementation with film formation solution led to decreasing the tensile strength and increasing the elongation of the gluten‐based films (Hammam, 2019). In general, the higher the lipid concentration, the lower the tensile strength value, which is in line with many previously reported studies (Shen et al., 2022; Zhang et al., 2018). This result can be attributed to the non‐polymeric nature of lipids, which inhibits their ability to form cohesive films, leading to the film's heterogeneous structure (Kowalczyk et al., 2016). Treatment 4 is an exception; The reason for this can be expressed by the negative effect of DATEM and beeswax in this ratio (50/50) on the gluten network.

The elongation at break decreased with the addition of DATEM (See Table 2). This decrease may be caused by solid particles that can cause the formation of a rigid dispersed phase in the film, in turn leading to decreased flexibility of the film (Shen et al., 2022). In contrast, the elongation at break increased with the incorporation of beeswax, suggesting that beeswax has a strong plasticizing effect on the films, causing the network structure of the film to be discontinuous (Valizadeh et al., 2019). Therefore, the highest amount of elongation at break was observed in treatment 6, which contains just beeswax. Similar results have also been previously observed (Nurul Syahida et al., 2020; Shen et al., 2022).

The characteristics of films made from wheat gluten extracted from four types of wheat flour have been investigated during a study in Brazil (Tanada‐Palmu & Grosso, 2003). The mechanical properties of the films were: tensile strength in four types of films 69, 58, 21, and 22 MPa; and the elongation at break in the films were 121%, 93%, 76%, and 19%. As can be seen, there is a significant difference between the mechanical properties of wheat gluten‐based film from different species. This difference can be attributed to the type of wheat, gluten extraction conditions, film formulation, drying and formation conditions of film, and the amount of plasticizer and working conditions with the device for measuring mechanical properties.

### 3.2 Water solubility of the film

Increasing lipids in gluten‐based films reduce the solubility of these films in water and this is due to the hydrophobicity of lipids (Shen et al., 2022). However, due to DATEM's hydrophilic nature, it reduces gluten‐based film solubility less than hydrophobic lipids (Avramescu et al., 2020). As shown in Table 2, in treatment 1, which contains gluten without lipids, the solubility is 20.60%. By adding DATEM, the solubility is reduced so that in treatment 2, which contains 100% DATEM, the solubility is 16.07%. By decreasing the percentage of DATEM and increasing the percentage of beeswax in the formula, the solubility decreased more, and finally, the lowest amount (13.33%) was observed in treatment 6 with 100% beeswax. Therefore, it can be concluded that the lipid‐free gluten‐based film had the highest water solubility, and the gluten‐based film containing just beeswax had the least water solubility. This result is consistent with previous investigations (Khanzadi et al., 2015; Shen et al., 2022; Zhang et al., 2018).

### 3.3 Water vapor permeability of the film

The addition of lipid components up to 20 g per 100 g of dry matter of the gluten‐based film, leads to a sharp decrease in the water vapor permeability of the film, and this is due to the presence of many non‐polar groups and their hydrophobicity that prevents the transfer of water molecules through the film (Shen et al., 2022). According to Table 2, treatment 1 (lipid‐free) had the highest water vapor permeability, treatment 6 (just beeswax) had the lowest water vapor permeability, and the rest of the treatments are in the middle. Beeswax is a hydrophobic lipid, and could thus effectively reject moisture and reduce hydrophilic interactions; thus, it could significantly decrease the water vapor permeability values of the gluten‐based films. In general, waxes normally are comprised of esters of fatty acids and long‐chain alcohols (Jannin & Cuppok, 2013). They do not possess any polar constituents or hydrophilic parts, indicating that they cannot interact with water. Therefore, waxes have very low water vapor permeability (Chen et al., 2021).

### 3.4 Color analysis of the film

Color is an important organoleptic parameter, and the success of coating it is represented by the final product color (Avramescu et al., 2020). The results obtained from the film color measurement (Table 3) for different treatments showed that the addition of lipids to the gluten‐based film increased the brightness (L*), which may be due to the color of these white compounds. Further increase in brightness with the increasing amount of DATEM was due to its white color. Beeswax is light yellow but lighter than gluten, which is yellowish‐brown. The results showed that gluten without lipids has the lowest amount of L* (83.23) and gluten with 100% DATEM has the highest amount of L* (85.57). However, the number of around 80 produced films indicates the good transparency of these films to cover food. The surface morphology attained during film drying determines how shiny the edible films will be. In general, the brightness of edible films increases with the smoothness of their surface (Zoghi et al., 2020).

**TABLE 3:** Color analysis of gluten‐based edible films

Treatment^A^	L*	a*	b*
T1	83.23 ± 0.01^a^	−0.83 ± 0.03^a^	25.36 ± 0.10^a^
T2	84.72 ± 0.04^b^	−1.26 ± 0.02^b^	24.20 ± 0.03^b^
T3	84.82 ± 0.21^b^	−1.32 ± 0.01^b^	24.12 ± 0.03^b^
T4	85.29 ± 0.05^c^	−1.41 ± 0.04^c^	22.59 ± 0.15^c^
T5	85.51 ± 0.16^cd^	−1.43 ± 0.02^cd^	22.52 ± 0.10^cd^
T6	85.57 ± 0.08^d^	−1.47 ± 0.15^d^	22.40 ± 0.04^d^

*Note*: Data are expressed as mean values. Values in the columns followed by different lowercase letters are significantly different (*p* < .05).

^A^ For treatment descriptions see Table 1.

The value of a* is negative in all treatments and this indicates the presence of green in all of them. By adding the DATEM and increasing its ratio in the formula, the green color increases, but the amount of a* in the lipid‐free gluten‐based film is the least (−0.83).

The value of b* is positive in all treatments, which indicates the presence of yellow in all of them. Most yellowness is related to lipid‐free gluten‐based film (25.36) but with the addition of beeswax and DATEM, the amount of b* was reduced. The greatest decrease was observed with increasing the DATEM ratio, so the film with 100% DATEM had the least yellowing (22.40). This is due to the white color of the DATEM and the light‐yellow color of the beeswax compared to the yellow color of the gluten. Millard reaction is one of the effective factors in the amount of b*. Also, the amount of yellow color of gluten varies in different types of wheat. Generally, factors affecting the color of edible films include the average size and crystallinity of the crystallites, the quantity and kind of plasticizer, as well as structural conformation, refractive index, and compatibility of the film compounds (Zoghi et al., 2020).

### 3.5 Hardness evaluation of coated bread

According to the results, treatments 2 and 6 (see Table 1) were selected as the optimum formula for coating bread samples. Because treatments 6 and 2 had the best characteristics in terms of water vapor permeability and mechanical properties, respectively. So, other experiments were done using a control sample (bread without coating), and bread samples with coating treatments 2 and 6.

Based on the data in Table 4, the hardness of bread coated with treatment 6 (just beeswax) on the 1st, 3rd, and 5th days of storage, in polyethylene bags, was significantly different from bread coated with treatment 2 (just DATEM) and bread without coating. Bread coated with treatment 6 showed the least hardness, and then bread coated with treatment 2 was placed, and the highest hardness was related to bread without coating. According to Table 5, the same result was obtained for the hardness of coated and uncoated bread samples without the use of polyethylene bags at 50% relative humidity. Since treatment 6 had lower water vapor permeability compared to treatment 2, these results were expected. The reason for this result is that less moisture was removed from the coated bread samples than from the uncoated ones and therefore, they become softer.

**TABLE 4:** Hardness and staling feature of coated hamburger bread samples on the first, third, and fifth day of storage time in polyethylene bags

Treatment^A^	Hardness (*N*)			Staling feature		
	First day	Third day	Fifth day	First day	Third day	Fifth day
Control	6.04 ± 0.11^a^	10.88 ± 0.68^a^	15.53 ± 0.86^a^	4.6 ± 0.52^a^	3.4 ± 0.52^a^	2.0 ± 0.67^a^
T2	4.94 ± 0.36^b^	8.30 ± 0.59^b^	11.43 ± 0.79^b^	4.8 ± 0.42^a^	4.1 ± 0.57^b^	3.2 ± 0.63^b^
T6	3.84 ± 0.23^c^	6.04 ± 0.50^c^	8.92 ± 0.23^c^	4.9 ± 0.57^a^	4.2 ± 0.42^b^	3.3 ± 0.67^b^

*Note*: Data are expressed as mean values. Values in the columns followed by different lowercase letters are significantly different (*p* < .05).

^A^ For treatment descriptions see Table 1.

**TABLE 5:** The hardness of hamburger bread samples on the first, second, and third day of storage time without a polyethylene bag

Treatment^A^	Hardness (*N*)		
	First day	Second day	Third day
Control	5.38 ± 0.19^a^	7.42 ± 0.22^a^	15.67 ± 0.47^a^
T2	4.55 ± 0.26^b^	5.67 ± 0.47^b^	12.81 ± 0.33^b^
T6	3.26 ± 0.08^c^	4.49 ± 0.22^c^	10.81 ± 0.67^c^

*Note*: Data are expressed as mean values. Values in the columns followed by different lowercase letters are significantly different (*p* < .05).

^A^ For treatment descriptions see Table 1.

One of the most crucial elements affecting bread hardness is the moisture content of the crumb. Reducing moisture enhances the hydrogen bonding between starch filaments and the development of links between protein and starch, which in turn promotes hardness because water can act as a softener in bread (Silva et al., 2016). Similar results have been obtained in this field, which shows the positive role of these coatings in reducing hardness (Balaguer et al., 2013; Chen et al., 2021; Heras‐Mozos et al., 2019). Bravin et al. (2006) prepared an edible coating of cornstarch, methylcellulose, and soybean oil and sprayed it on crackers. Their results showed that at a relative humidity of 65% the shelf life of coated crackers was 42 h and of uncoated crackers for 27 h.

### 3.6 Sensory assessment of coated bread

Considering the mean scores of sensory tests of coated and uncoated bread samples on days 1, 3, and 5 of storage (Table 4), it can be concluded that on the first day there was no significant difference between the three samples; and on the third and fifth days, there was no significant difference between the two coated bread samples, but there was a significant difference between them and bread without coating. Significant differences between coated bread samples and uncoated bread are consistent with the hardness evaluation test results and show less staleness in coated bread samples. Staleness is a process that reduces the acceptability of baking industry products by consumers and includes changes in the core of bread.

Generally, staleness is characterized by leathery bread crust, hardening of bread core, decrease in moisture and taste, and decrease in freshness in the product (Silva et al., 2016). During the bread's storage period, complicated molecular structure changes cause the staling phenomena to take place. Bread's freshness is negatively impacted by water loss during the shelf life, which causes earlier staling (Kim & Yoo, 2020). Therefore, using edible coatings could help delay staling in hamburger bread. Chen et al. (2021) studied a wide range of edible coating ingredients, including beeswax, gum, starch, protein, and cellulose derivatives to control bread staling. According to their results, all wax coatings could decrease bread moisture loss, crumb hardening, and retard bread staling significantly (Chen et al., 2021).

### 3.7 Evaluation of mold growth in coated bread

Due to the addition of sorbate as a preservative and the use of ethanol in the composition of the coatings, mold growth was not observed in any of the coated and uncoated bread samples during the 1st, 3rd, and 5th days of storage. Several authors have reported that the use of coating can reduce the incidence of mold infections in bread (Balaguer et al., 2013; Heras‐Mozos et al., 2019; Passarinho et al., 2014). Heras‐Mozos et al. (2019) developed films containing garlic extract to apply as a coating for the preservative‐free sliced pan loaf, to extend its shelf‐life. They stated that control samples showed the first symptoms of mold growth on day 5, but in the case of film‐coated with ethylene‐vinyl alcohol copolymer or polyethylene containing 0.5% of garlic extract, mold growth began on days 9 and 12, respectively.

## 4 CONCLUSIONS

In this work, a gluten‐based film containing lipids, beeswax and/or DATEM, was successfully applied for the coating of hamburger bread in order to reduce staling process and improve quality. According to the obtained results, the effects of lipids addition on the mechanical and water barrier properties of the films were dependent upon the content and type of lipids. Adding DATEM or beeswax, separately, to the gluten film formulae reduced the solubility and water vapor permeability of the film, especially in the case of beeswax. Furthermore, the presence of lipids decreased tensile strength and elongation at the break of the film. Using gluten‐DATEM or gluten‐beeswax coatings for hamburger bread reduced the hardness of the bread and delays its staleness. Overall, the results of the sensory evaluation showed more acceptability for coated bread samples because of their softer crust, due to the good mechanical properties of the coating. More mechanism studies on the index of the film, such as Fourier infrared and XRD; and more investigations on the bread texture indicators, such as chewiness, are needed. Also, further research should focus on the incorporation of other types of lipids at different levels into the gluten‐based films/coatings and the evaluation of applying these coatings onto the other types of bread.

## CONFLICT OF INTEREST

The authors declare no conflicts of interest.

## ETHICS STATEMENT

We declare no ethical issue related to this article.

## Data Availability

Data sharing is not applicable to this article.
